# Effects of Orem's Self-Care Model on the Life Quality of Elderly Patients with Hip Fractures

**DOI:** 10.1155/2020/5602683

**Published:** 2020-05-20

**Authors:** Xiaodong Xu, Jun Han, Yajia Li, Xichun Sun, Peng Lin, Ying Chen, Fuqiang Gao, Zirong Li, Shuai Zhang, Wei Sun

**Affiliations:** ^1^Department of Orthopedics, China-Japan Friendship Hospital, Beijing 100029, China; ^2^Department of Orthopedic Surgery, Peking University China-Japan Friendship School of Clinical Medicine, Beijing 100029, China; ^3^Department of Dermatology, Xiangya Hospital, Central South University, Changsha 410008, China; ^4^Department of Orthopedics, People's Hospital of Ningxia Hui Autonomous Region, Yinchuan 750001, China; ^5^Department of Neurology, Affiliated Hospital of Yangzhou University, Yangzhou University, Hanjiang Road, Yangzhou 225100, China

## Abstract

**Background:**

Hip fractures of elderly patients are a public health problem worldwide, mostly lying in bed for a long time; therefore, the importance of life quality in such patients is an issue beyond question. Orem's self-care model is a nursing pattern which is introduced with the purpose of improving the self-care ability of individuals, especially the patients suffering from diseases with limits on activity.

**Objective:**

The aim of this study was to determine the effects of Orem's self-care program on life quality of senile patients with hip fractures.

**Methods:**

A randomized clinical trial study was conducted on 130 eligible old patients suffering from hip fractures who were selected using easy sampling methods and allocated randomly into two groups of experiment and control. The data were collected through validated questionnaires including visual analogue scale (VAS) and Barthel index for them. The experiment group was treated according to Orem's self-care model, and the control group was treated on the basis of the traditional care model. The data of complications including pneumonia, deep venous thrombosis, urinary infection, wound problem, and bedsore were also gathered.

**Results:**

As revealed, mean scores of VAS and Barthel index one week after operation in the experiment group were significantly different from the control one (*P* < 0.05, *P* ≤ 0.001). The changes of VAS and Barthel index six weeks postoperatively of the two groups were also statistically significant (*P* < 0.05, *P* ≤ 0.001). Compared with the control group, the difference of complications reduced significantly in the experiment group (*P* < 0.05). Accordingly, educational intervention according to Orem's self-care model seemed to be effective in promoting self-care ability for these senile patients.

**Conclusions:**

According to the obtained results, a self-care program based on Orem's model for elderly patients with hip fractures can improve life quality and reduce perioperative complications significantly. Therefore, it is recommended that this nursing program should be taken into account as a part of treatment measures for these patients.

## 1. Introduction

Fractures due to fragility of the bone around hip joints have become a major public health issue, presenting with an increasing incidence due to the growth of elderly population [1]. The annual incidence of hip fractures worldwide is estimated to increase from 1.6 million in 2000 to at least 4.5 million by 2050 due primarily to population aging [2]. Nowadays, hip fractures have become a uniquely challenging global health problem with significant socioeconomic consequences and health care budgets, with considerable risks of dependence in activities of daily living, complications, and mortality [3, 4]. Many healthy and active senile individuals suffering from hip fractures lose their independent mobility, whereas the weaker patients may lose their ability of independent living at home; the weakest patients with already-distressing health status become further debilitated by pain, anxiety, lying in bed, and inability to take care of themselves [5, 6]. Also, a lot of physical, psychological, social, and economic complications occurring in the perioperative period, such as pneumonia, deep venous thrombosis and pulmonary embolus, urinary retention, wound problem, pressure ulcers, and pain have been reported to have negative impacts on patients [7]. They undergo considerable difficulties in returning to their prefracture living situation and in achieving full recovery of function and living quality [8]. Although nursing teams provide them with information about treatment measures and complications in the form of brochures, the initial surveys and interviews with patients and their families have shown that they seriously need to learn more about severity degree of the fracture, specific operation methods, complications and possible preventive measures, and self-care knowledge [9]. Orem's theory was presented in 1959, and the application of this self-care model has had positive effects on patients with different diseases [10–13]. However, we found no studies that applied this model to orthopedic patients such as those with hip fractures. Therefore, the present study aims to examine the effects of educational interventions according to Orem's self-care theory on the life quality of elderly patients with hip fractures including femoral neck fractures and intertrochanteric fractures.

## 2. Patients and Methods

### 2.1. Trial Design and Participants

This study was a randomized clinical trial in which the effect of application of Orem's self-care program on life quality of elderly patients with hip fractures admitted in the orthopedics departments had been investigated. Two groups of patients were selected and randomly assigned to experiment and control groups.

130 patients were enrolled in the study including 65 patients for the experiment group and 65 patients for the control one. Experiment group comprised 30 cases of femoral neck fractures and 35 cases of intertrochanteric fractures, with an average age of 77.6 years old (Table 1). Control group consisted of 29 cases of femoral neck fractures and 36 cases of intertrochanteric fractures (Table 1), whose average age was 76.6 years old.

### 2.2. Selection Criteria

The inclusion criteria were as follows: (1) over 60 years of age; (2) isolated unilateral hip fractures including femoral neck fractures and intertrochanteric fractures; (3) fresh closed fractures (<14 days); (4) the vital signs and hemodynamics were stable; (5) no diagnosis of pneumonia, deep venous thrombosis, urinary infection, wound problem, and bedsore when admitted to the hospital; (6) be able to communicate face to face normally; and (7) undergoing the orthopedic surgery for the first time.

Exclusion criteria consisted of failure to complete the consent forms, giving up the study, and inability to participate in all the educational sessions with varying degrees of disturbance of consciousness and mental changes.

### 2.3. Instrument Design

Data collection tool was a valid and reliable questionnaire based on Orem's self-care model including visual analogue scale (VAS) and Barthel index. Visual analogue scale is a simple and frequently used method for assessment of variations in intensity of pain. VAS is designed to present to the respondent a rating scale with minimum constraints. Respondents mark the location on the 10-centimeter line corresponding to the amount of pain they experienced. This gives them the greatest freedom to choose their pain's exact intensity. It also gives the maximum opportunity for each respondent to express a personal response style [14]. Barthel index is another tool used in the study (Table 2). The Barthel index is a scale that measures disability or dependence in activities of daily living of patients including ten indices: feeding, bathing, grooming, dressing, bowel control, bladder control, toilet use, transfers, mobility, and stairs. The Barthel index is scored from 0 to 100, with 5-point increments. We considered anyone with a score <100 as having some disability [15]. Barthel index is a very simple tool and can be easily administered by the health care professional [16]. Informed consent was obtained from all patients participating in the study.

### 2.4. Interventions and Data Collection

Patients were randomly allocated to either the experiment group or the control one. Patients in the experiment group received education, support, and counseling on the basis of Orem's model, while patients in the control group received no intervention except the traditional routine orthopedic nursing care. Once we confirmed the experiment and control groups, we must obtain their consent forms and ensure that no significant difference existed between them in terms of demographic features, and Orem's model-based self-care questionnaires were completely filled. The educational content was prepared based on provided data by the participants and literature reviews. The experiment group received education in self-care based on Orem's model until six weeks after operation. The educational program included oral interpretation, action modeling, and distributing educational package. For different periods of different patients, we developed different nursing plans, including wholly compensatory nursing, partially compensatory nursing, and supportive education, to provide a number of special nursing care interventions. These measures consisted of making an individualized brochure of health education to promote the knowledge of fracture, setting up a schedule for less pain, relieving the patient's anxiety by listening and providing mental support, and enhancing the patient's knowledge about the ability to control their discomfort. Educational sessions were held every day during the stay in the hospital, and guiding patients by using telephone was continued after leaving the hospital. Partial specific measures on the base of Orem's model against complications are shown in Table 3. One week and six weeks after surgery, Orem's model-based self-care questionnaires were filled by the two groups, respectively. The data of complications including pneumonia, deep venous thrombosis, urinary infection, wound problem, and bedsore were also collected, and the data were compared.

### 2.5. Statistical Analysis

Statistical analyses were performed with SPSS 20.0 for Windows (SPSS Inc., Chicago, IL, USA). All data are presented as mean ± standard deviation (SD). Differences between two groups were examined using an independent samples *t*-test in quantitative data. Chi-square tests were used to compare differences between groups in categorical data. A *P* value <0.05 was considered statistically significant.

## 3. Results

In Table 1, the self-care model components in the two groups were compared using independent samples *t*-test or Chi-squared test. As revealed, no significant difference was evident prior to intervention in terms of gender (*P*=0.551), age (*P*=0.197), BMI (*P*=0.120), and fracture type (*P*=0.878), respectively.

Also, data analysis of one week after operation showed that values of VAS in experiment and control groups were 4.4 ± 1.6 and 4.8 ± 1.9, and Barthel index of the two groups was 47.2 ± 11.9 and 43.4 ± 13.3, respectively (Table 4). The difference of two indicators in the two groups was statistically significant. And six weeks after operation, VAS and Barthel index in the experiment group were 1.9 ± 0.9 and 86.2 ± 12.8, while the control one showed 2.4 ± 1.5 and 81.3 ± 11.9. Mean scores of VAS and Barthel index one week after operation in the experiment group were significantly different from the control one (*P*=0.009, *P* ≤ 0.001, respectively). The changes of VAS and Barthel index six weeks after operation of the two groups were also statistically significant (*P*=0.016, *P* ≤ 0.001) (Table 4).

The patients suffering from pneumonia, deep venous thrombosis, urinary infection, wound problem, and bedsore were 4, 2, 1, 2, 1 and 8, 5, 2, 2, 2 in the experiment and control groups, respectively. There was a statistically significant difference of complications between the two groups (Table 5).

## 4. Discussion

Femoral neck fractures and intertrochanteric fractures are the most common types of hip fragility fractures, which are related to osteoporosis and have a dramatic influence on the elderly people, and they are associated with excess mortality and morbidity resulting in costly hospital and lengthy rehabilitation procedures. These patients experience considerable difficulties in returning to their prefracture living situation and in achieving full recovery of function [8]. Due to the low life quality of these patients, taking some measures to improve survival quality is quite essential [17]. Orem's self-care theory can help health care providers identify and satisfy patients' self-care needs since it has been proved that self-care could show immeasurable potential and practicability on the theory and practice [13]. With the socialization and familiarization of the current nursing services, self-care is becoming a developing trend nowadays. The patients necessitate long-term self-care skills, so teaching people the knowledge and technology will be a new requirement for nursing. It is required that doctors and nurses should pay attention to cultivating patients' self-care ability, mobilizing their subjective initiative, and promoting patients to take the responsibility of self-care.

The present study is the first to adopt Orem's self-care nursing theory to investigate the effects of educational intervention of aged patients of hip fractures. The traditional orthopedic routine nursing program with many deficiencies does not take into account the self-care ability of patients since nursing measures are dogmatic, and patients are accustomed to passive nursing. As a result, the nursing measures for patients who cannot take care of themselves are not very effective, which are not conducive to the rehabilitation. However, according to the specific patients and disease conditions, the self-care theory dynamically evaluates their self-care ability, adopts different nursing measures, and formulates the self-care model suitable for different individuals and stages. Moreover, nursing at all levels can be valued, and the corresponding compensation measures can be obtained according to their self-care ability. The supportive educational intervention can improve their self-care ability in and out of hospital and obviously affect public health outcomes [13].

The changes of VAS and Barthel index six weeks after operation of the two groups were also statistically significant (*P*=0.016, *P* ≤ 0.001) since patients in the experiment group had higher levels of self-care knowledge, motivation, and skills compared with the control one. All the patients of the experiment group were provided counseling services and began to participate in their nursing decisions from the time of admission. The measures encouraged them and their family members to participate in nursing activities together, mobilized their enthusiasm, explored their self-care potential, and maximized their self-care ability. It enabled the patients to correctly understand the diseases and master relevant self-care knowledge, so as to reduce the occurrence of complications and improve hip function postoperatively. Also, we made great efforts to enhance the patients' confidence, enabled them to correctly treat and accept the changes in the internal and external environment, and improved the living quality. At the same time, it enhanced the communication and understanding between medical staff and patients and promoted the harmonious development of our relationship. The results showed that designing and implementing a training program based on Orem's theory can be effective in satisfying patients' requirements and improving their life quality.

Partial specific measures according to Orem's theory were formulated and implemented in the experiment group. Detailed nursing interventions were performed which included different measures to prevent pneumonia, deep venous thrombosis, urinary infection, wound problem, and bedsore (Table 3). Wholly compensatory nursing, partially compensatory nursing, and supportive education were implemented depending on the patient's condition. According to our study, compared with the control group, the difference of complications reduced significantly in the experiment group (*P*=0.033). Complications may occur during as many as 20% of all hospitalizations for hip fractures, and a few postfracture complications are potentially modifiable [18]. Different nursing decisions may influence the care of these frail elders, so high-quality nursing may have less pneumonia and delirium. These complications may be modifiable; nursing is so important to cause marked variations in the incidence such as pneumonia and pressure ulcer. Aggressive preventive measures and skin management strategies might reduce the complications among patients with hip fractures and ultimately lead to improve survival of the vulnerable population [19]. Therefore, educational intervention according to Orem's self-care model seems to be effective in decreasing complications.

Therefore, a comprehensive, well-designed, and appropriate program based on Orem's model can decrease discomfort and complications, improve quality of care, help the patients to reduce dependency, and achieve the optimal health. The Orem-based self-care model program along with routine nursing care can be a useful tool to improve life quality in hip fractures. Thus, medical staff can design and train a self-care program as a part of the therapeutic process and prevent a lot of mental and psychosocial problems [8, 20].

There are limitations remained in this study. First, the sample size was relatively limited, especially the subgroups; a larger sample size might be better to find a significant difference between different groups. Second, despite the selection of variables and data collection tools which was considered and justified based on the extensive literature review, there might have been other important variables or tools which have not been selected for this study. Third, there was also one limitation due to the clinical condition that the follow-up time was not so long, which should be researched in the future.

## 5. Conclusion

Generally, a self-care program based on Orem's model for elderly patients with hip fractures can improve life quality and reduce perioperative complications significantly. The result was owing to the careful design and implementation of appropriate care measures catering to the needs, interests, individualism, and problems of these patients. An educative and supportive nursing system is feasible and useful in hip fractures. Therefore, designing a self-care nursing program based on Orem's self-care model should be considered to reduce complications and problems closely related to hip fragility fractures.

## Figures and Tables

**Table 1:** Baseline characteristics of patients in the two groups.

	Variables	Exp.	Con.	*P* value
Gender	M.	24	27	0.551
	F.	41	38	

Age		77.6 ± 4.8	76.6 ± 4.1	0.197

BMI		23.9 ± 2.9	24.1 ± 3.6	0.120

Fracture type	Neck.	30	29	0.878
	Inter.	35	36	

Exp., experiment group; Cont., control group; M., male; F., female; BMI, body mass index; Neck., femoral neck fractures; Inter., femoral intertrochanteric fractures.

**Table 2:** Barthel index: rank the patient's independence in the following areas.

Feeding	Independent	10
	Needs help	5
	Unable	0

Bathing	Independent	5
	Unable	0

Grooming	Independent	5
	Unable	0

Dressing	Independent	10
	Needs help	5
	Unable	0

Bowel control	Continent	10
	Occasional accident	5
	Incontinent (or needs to be given enemas)	0

Bladder control	Continent	10
	Occasional accident	5
	Incontinent (catheterized, unable to manage alone)	0

Toilet use	Independent	10
	Needs help	5
	Unable	0

Transfers (bed to chair and back)	Independent	15
	Needs minor help (verbal or physical)	10
	Needs major help (1-2 people, physical), can sit	5
	Unable	0

Mobility on level surfaces	Independent (but may use any aid, e.g., stick), >50 yards	15
	Walks with help of one person (verbal or physical), >50 yards	10
	Wheelchair independent, including corners, >50 yards	5
	Immobile or <50 yards	0

Stairs	Independent	10
	Needs help (verbal, physical, carrying aid)	5
	Unable	0

**Table 3:** Partial specific measures against complications of wholly compensatory nursing, partially compensatory nursing, and supportive educative nursing in the experiment group.

Against pneumonia	Systematic respiratory function exercise; encouraging deep breathing; effective coughing up phlegm; blowing a balloon or application of breath training devices; sitting up more and earlier; back-patting for sputum excretion; aerosol inhalation.

Against deep venous thrombosis	Counseling and encouraging; observing the swelling and pain of the limbs; replenishing blood volume appropriately such as drinking more water; monitoring clotting function; application of painkillers; anticoagulant drugs; continuous active motion of the lower limbs; physical measures (continuous passive motion; intermittent pneumatic compression; venous foot pump; graduated compression stockings); going to the ground earlier.

Against urinary infection	No catheterization for short operation time; drinking more water; urethral mouth care on time; strict asepsis procedure of catheterization; keeping the catheter unobstructed; bladder function exercise before withdrawal of the catheter; pulling out the catheter as soon as possible; fomenting the lower abdomen.

Against wound problem	Cold compresses; covering wound with dressing completely; keeping wound dressing dry and clean; disinfection completely and changing fresh dressing on time; encouraging a high-protein diet; proper application of antibiotics.

Against bedsore	Antidecubitus mattress; defecation care and keeping clothes and skin clean; changing the position frequently; turnover on time; covering with soft dressing; doing local massage; physical therapy.

**Table 4:** Comparison of VAS and Barthel index one week and six weeks after operation in both experiment and control groups.

	VAS 1	Barthel 1	VAS 2	Barthel 2
Exp.	4.4 ± 1.6	47.2 ± 11.9	1.9 ± 0.9	86.2 ± 12.8
Con.	4.8 ± 1.9	43.4 ± 13.3	2.4 ± 1.5	81.3 ± 11.9
*P* value	0.009	≤0.001	0.016	≤0.001

Exp., experiment group; Cont., control group; VAS 1, VAS of one week after operation; VAS 2, VAS of six weeks after operation; Barthel 1, Barthel index of one week after operation; Barthel 2, Barthel index of six weeks after operation.

**Table 5:** Comparison of complications in both experiment and control groups.

Complication	Exp.	Con.	*P* value	*X* ^2^
Pneumonia	4	8	0.363	0.826
Deep venous thrombosis	2	5	0.437	0.604
Urinary infection	1	2	—	—
Wound problem	2	2	—	—
Bedsore	1	2	—	—
Total	9	19	0.033	4.552

Exp., experiment group; Cont., control group.

## Data Availability

All data included in this study are available upon request by contacting with the corresponding authors.

## References

[1] Alexiou K., Roushias A., Varitimidis S., Malizos K. (2018). Quality of life and psychological consequences in elderly patients after a hip fracture: a review. *Clinical Interventions in Aging*.

[2] Gullberg B., Johnell O., Kanis J. A. (1997). World-wide projections for hip fracture. *Osteoporosis International*.

[3] Marques A., Lourenço Ó., da Silva J. A. P. (2015). The burden of osteoporotic hip fractures in Portugal: costs, health related quality of life and mortality. *Osteoporosis International*.

[4] Wiklund R., Toots A., Conradsson M. (2016). Risk factors for hip fracture in very old people: a population-based study. *Osteoporosis International*.

[5] Hallberg I., Rosenqvist A. M., Kartous L. (2004). Health-related quality of life after osteoporotic fractures. *Osteoporosis International*.

[6] Peterson M. G. E., Allegrante J. P., Allegrante J. P. (2002). Measuring recovery after a hip fracture using the SF-36 and cummings scales. *Osteoporosis International*.

[7] McLaughlin M. A., Orosz G. M., Magaziner J. (2006). Preoperative status and risk of complications in patients with hip fracture. *Journal of General Internal Medicine*.

[8] Fierens J., Broos P. L. O. (2006). Quality of life after hip fracture surgery in the elderly. *Acta Chirurgica Belgica*.

[9] Hashemi F., Rahimi Dolatabad F., Yektatalab S., Ayaz M., Zare N., Mansouri P. (2014). Effect of orem self-care program on the life quality of burn patients referred to Ghotb-al-Din-e-Shirazi burn center, Shiraz, Iran: a randomized controlled trial. *International Journal of Community Based Nursing and Midwifery*.

[10] Khatiban M., Shirani F., Oshvandi K., Soltanian A. R., Ebrahimian R. (2018). Orem’s self-care model with trauma patients: a quasi-experimental study. *Nursing Science Quarterly*.

[11] Afrasiabifar A., Mehri Z., Sadat S. J., Shirazi H. R. G. (2016). The effect of orem’s self-care model on fatigue in patients with multiple sclerosis: a single blind randomized clinical trial study. *Iranian Red Crescent Medical Journal*.

[12] Sharifi N., Majlessi F., Montazeri A., Shojaeizadeh D., Sadeghi R. (2017). Prevention of osteoporosis in female students based on the Orem self-care model. *Electronic Physiciane*.

[13] Mohammadpour A., Rahmati Sharghi N., Khosravan S., Alami A., Akhond M. (2015). The effect of a supportive educational intervention developed based on the Orem’s self-care theory on the self-care ability of patients with myocardial infarction: a randomised controlled trial. *Journal of Clinical Nursing*.

[14] Aitken R. C. B. (1969). A growing edge of measurement of feelings [abridged]. *Proceedings of the Royal Society of Medicine*.

[15] Collin C., Wade D. T., Davies S., Horne V. (1988). The Barthel ADL index: a reliability study. *International Disability Studies*.

[16] Gupta S., Yadav R., Malhotra A. (2016). Assessment of physical disability using Barthel index among elderly of rural areas of district Jhansi (U.P), India. *Journal of Family Medicine and Primary Care*.

[17] Østhus T. B. H., Von Der Lippe N., Ribu L. (2012). Health-related quality of life and all-cause mortality in patients with diabetes on dialysis. *BMC Nephrology*.

[18] Berry S. D., Samelson E. J., Bordes M., Broe K., Kiel D. P. (2009). Survival of aged nursing home residents with hip fracture. *The Journals of Gerontology Series A: Biological Sciences and Medical Sciences*.

[19] Roche J. J. W., Wenn R. T., Sahota O., Moran C. G. (2005). Effect of comorbidities and postoperative complications on mortality after hip fracture in elderly people: prospective observational cohort study. *BMJ*.

[20] Adib Hajbaghery M., Abbasinia M. (2013). Quality of life of the elderly after hip fracture surgery: a case-control study. *Journal of Caring Sciences*.

